# Autophagy Induced Accumulation of Lipids in *pgrl1* and *pgr5* of *Chlamydomonas reinhardtii* Under High Light

**DOI:** 10.3389/fpls.2021.752634

**Published:** 2022-01-25

**Authors:** Nisha Chouhan, Elsinraju Devadasu, Ranay Mohan Yadav, Rajagopal Subramanyam

**Affiliations:** Department of Plant Sciences, School of Life Sciences, University of Hyderabad, Hyderabad, India

**Keywords:** *Chlamydomonas reinhardtii*, cyclic electron transport, lipid bodies, Nile Red fluorescence, triacylglycerol

## Abstract

*Chlamydomonas (C.) reinhardtii* is a potential microalga for lipid production. Autophagy-triggered lipid metabolism in microalgae has not being studied so far from a mutant of proton gradient regulation 1 like (PGRL1) and proton gradient regulation 5 (PGR5). In this study, *C. reinhardtii* cells (wild-type CC124 and cyclic electron transport dependant mutants *pgrl1* and *pgr5*) were grown photoheterotrophically in high light 500 μmol photons m^–2^ s^–1^, where *pgr5* growth was retarded due to an increase in reactive oxygen species (ROS). The lipid contents were increased; however, carbohydrate content was decreased in *pgr5*. Further, the Nile Red (NR) fluorescence shows many lipid bodies in *pgr5* cells under high light. Similarly, the electron micrographs show that large vacuoles were formed in high light stress despite the grana stacks structure. We also observed increased production of reactive oxygen species, which could be one reason the cells underwent autophagy. Further, a significant increase of autophagy ATG8 and detections of ATG8-PE protein was noticed in *pgr5*, a hallmark characteristic for autophagy formation. Consequently, the triacylglycerol (TAG) content was increased due to diacylglycerol acyltransferases (DGAT) and phospholipid diacylglycerol acyl-transference (PDAT) enzymes’ expression, especially in *pgr5*. Here the TAG synthesis would have been obtained from degraded membrane lipids in *pgr5*. Additionally, mono, polyunsaturated, and saturated fatty acids were identified more in the high light condition. Our study shows that the increased light induces the reactive oxygen species, which leads to autophagy and TAG accumulation. Therefore, the enhanced accumulation of TAGs can be used as feedstock for biodiesel production and aqua feed.

## Introduction

Light is vital for microalgae for efficient photosynthesis. CO_2_ fixation by Calvin Benson cycle occurs through photosynthesis that primarily synthesizes carbohydrates, leading to the synthesis of lipid stored as triacylglycerols (TAG) (Mondal et al., 2017). Microalgal species do not accumulate increased amounts of neutral lipids under normal growth conditions. Neutral lipid is accumulated under unfavorable conditions like nutrient, light, salt, and temperature stresses. Under normal light conditions, the rate of light absorbed is equal to the pace of photosynthesis, but when light intensity increases, the system cannot tolerate over-excitation. In this condition, the primary by-product of photosynthesis formed as reactive oxygen species (ROS), which regulate the autophagy (ATG) mechanism. Autophagy is a stress-responsive mechanism that can induce organelle deterioration (Liu and Bassham, 2012). This mechanism in *Chlamydomonas reinhardtii* regulates the degradative process in photosynthetic organisms. Autophagy plays a divulged role in the control of lipid metabolism. Inhibition of Target of Rapamycin (TOR) kinase by treatment of *C. reinhardtii* cells with rapamycin resulted in increased ATG8 lipidation (Pérez-Pérez et al., 2010) and vacuolization (Pérez-Pérez et al., 2010). Previous results have proven that ROS is an inducer of autophagy in algae (Pérez-Pérez et al., 2010). High light stress induces photo-oxidative damage due to ROS production, leading to autophagy activation in *C. reinhardtii* (Pérez-Pérez et al., 2010). A recent report has shown that a starchless mutant of *C. reinhardtii* induces oxidative stress, triggering autophagy, leading to TAG accumulation under nitrogen starvation (Pérez-Pérez et al., 2012). In a group of ATG (autophagy-related) proteins, the accumulation of ATG8 and ATG3 increased in a conditional repression line of a chloroplast protease (ClpP1), suggesting the chloroplast proteolysis systems and autophagy partially complement each other in *C. reinhardtii* (Ramundo et al., 2014). Additionally, these proteins were shown a similar function to that of other organisms (Pérez-Pérez et al., 2010, 2016). Loss of cytoplasmic structure with a significant increase in the volume occupied by lytic vacuoles and discharge of vacuole hydrolases can be assigned as autophagic cell death markers (Van Doorn, 2011). However, the mechanism of autophagy caused by high light stress in *C. reinhardtii* is poorly understood.

Additionally, it can achieve neutral lipid accumulation by exposing the cells to unfavorable conditions, such as removing the nutrients like nitrogen, sulfur, iron, or phosphate, or changing salinity and temperature (Alishah Aratboni et al., 2019). Most of the knowledge on TAG metabolism in *C. reinhardtii* has been gained from the N starvation (Siaut et al., 2011; Abida et al., 2015). Recent studies showed that cells exposed to small chemically active compounds could also accumulate TAG in *C. reinhardtii* (Wase et al., 2019). However, an increase in light intensity influences the microalgal lipid production in *Scenedesmus* at 6,000 lux (Mandotra et al., 2016), as well as *Nannochloropsis* sp at 700 μE m^–2^ s^–1^ (Pal et al., 2011) and *Botryococcus* sp. at 6,000 lux (Yeesang and Cheirsilp, 2011). Further, *Haematococcus pluvialis* (Zhekisheva et al., 2002), *Tichocarpus crinitus* (Khotimchenko and Yakovleva, 2005), and *Synechocystis* sp. (Cuellar-Bermudez et al., 2015) also showed an increased neutral lipid content under high light conditions.

Lipid bodies are synthesized in algal chloroplast by fatty acid synthase complex. These newly synthesized free fatty acids are translocated to the endoplasmic reticulum (ER), where they convert glyceraldehyde 3- phosphate (G3P) to diacylglycerol (DAG) through various enzymes. DAG is converted to triacylglycerol by the diacylglycerol acyltransferases (DGAT) enzyme. So, the enzyme is considered to reinforce the assembly of lipids in microalgae. DGAT enzyme catalyzes the TAG synthesis pathway’s ultimate step, and phospholipid diacylglycerol acyl-transference (PDAT) also helps accumulate TAG. However, it does not hook into the acyl CoA pathway. Here, PDAT transfers carboxylic acid moiety from a phospholipid to DAG to make TAG (Sorger and Daum, 2002; Shockey et al., 2006). A recent report from our group shows that a significant accumulation of TAG was observed under severe iron deficiency, and this could have been obtained from degraded chloroplast lipids through the DGAT enzyme (Devadasu et al., 2021).

Few studies have focused on increased fatty acid content, decreased photosynthetic activity, and retarded biomass, ultimately inhibiting microalgae growth in nutrient limitation. *pgrl1* is required for efficient cyclic electron transport (CET) and adenosine triphosphate (ATP) supply for efficient photosynthesis (Tolleter et al., 2011; Leister and Shikanai, 2013; Shikanai, 2014; Steinbeck et al., 2015). A *pgr5* mutant has been characterized in *C. reinhardtii* (Johnson et al., 2014), which revealed that *pgr5* deficiency results in a diminished proton gradient across the thylakoid membrane accompanied by less effective CET capacity. The CET pathway has been suggested to provide ATP for lipid production during N starvation in *C. reinhardtii* mutants impaired in *pgrl1*, which accumulates significantly fewer neutral lipids. However, under the high light condition, there is an increase in oil content after the 3rd day. So far, the accumulation of lipids has been well characterized in nutrient stress; however, not many studies are available under high light conditions. In *C. reinhardtii* (WT) the solar light is transformed into chemical energy using linear electron transport. However, *pgrl1* and *pgr5*, may show an increase in stromal redox poise that would cause an increase in lipid production under strong light.

In this study model, algae *C. reinhardtii* was propagated with different light conditions (50 and 500 μmol photons m^–2^ s^–1^) to realize insights into the autophagy and lipid accumulation response to high light. We propose that *C. reinhardtii* cells exposure to high light resulted in ROS accumulation, which induced autophagy and lipid accumulation in *pgr5*.

## Materials and Methods

### Strains and Culture Conditions

The green microalgae *C. reinhardtii* wild-type (WT) strain CC124 (137c) was obtained from the *C. reinhardtii* resource center (University of Minnesota) and mutants *pgrl1* and *pgr5* (137c background; Johnson et al., 2014), a kind of gift from Prof. Gilles Peltier (CEA-CNRS- Aix Marseille University, France) and Prof. Michael Hippler (University of Munster, Germany). Cells were grown photoheterotrophically in Tris-acetate phosphate (TAP) medium at 25°C with a photon flux density of 50 μmol photons m^–2^ s^–1^ in 250 mL conical flask shaken at 120 rpm in an orbital shaker at 25°C. The seed cultures of *C. reinhardtii* (WT, *prgl1*, and *prg5*) were harvested at an optical density (OD) of 0.8 and inoculated at OD of 0.02 for all the cultures. The cultures were cultivated photoheterotrophically at 25°C for 4 days at a continuous light intensity of 50 and 500 μmol photons m^–2^ s^–1^. Later the cultures were collected after the 3rd day with an OD of 0.8 at 750 nm. Light intensity was measured and adjusted with a light meter (Hansatech).

### Biomass Determination

To calculate the total biomass of cells (WT and mutants *pgrl1* and *pgr5*), 5 mL of culture was collected after the 3rd day of growth in high light and control by centrifugation at 1,000 × *g* for 10 min at room temperature (RT), and the supernatant was discarded. The excess media was removed by short centrifugation, and cell pellets were lyophilized at −109°C for 12 h. The cell pellet was washed with distilled water and transferred into a pre-weighed 15 mL falcon tube. The total dry weight was later quantified by subtracting the empty tube.

### Reactive Oxygen Species Measurement

The total ROS was detected with 2,7-dichlorodihydrofluorescein diacetate (H_2_DCFDA) (Sigma-Aldrich), a fluorescence dye used to see the ROS in live *C. reinhardtii* cells. Cells at a 3 million density were collected from all the conditions, and H_2_DCFDA staining was performed at 10 μM concentration (Upadhyaya et al., 2020). Further, cells were incubated with dye for 1 h at RT in a continuously rotating shaker under dark. Images were captured using Carl Zeiss NL0 710 Confocal microscope. H_2_DCFDA was detected in a 500–530 nm bandpass optical filter with an excitation wavelength of 492 nm and an emission wavelength of 525 nm. Chlorophyll auto-fluorescence was detected using an optical filter of 600 nm. Samples were viewed with a 60× oil immersion lens objective by using the ZEN, 2010 software.

Total ROS produced by *C. reinhardtii* cells was also quantified by spectrophotometer. Logarithmic-phase cells were centrifuged at 1,500 × *g*, 20°C for 5 min, resuspended in an equal volume of fresh TAP medium, and then incubated in 5 μM DCFH-DA for 1 h in the dark at 25°C. After dark exposure, the cells were washed twice with TAP to remove excess dye (Devadasu et al., 2019). The fluorescence was measured using a Microplate reader (Tecan M250) at an excitation of 485 nm and an emission of 530 nm. We calculated the total ROS by subtracting the value of fluorescence with dye and without dye.

### Transmission Electron Microscopy

The cells (3 × 10^6^ cells/ml) were resuspended in 0.1 M phosphate buffer (pH 7.2). Cells were fixed in a 2% Glutaraldehyde (Sigma-Aldrich, St. Louis, United States) solution for 2 h in the dark. The cells were then washed four times in PBS buffer for 1 h each time before being post-fixed in 2% aqueous Osmium Tetraoxide for 3 h, washed six times in deionized distilled water, dehydrated in a series of ethanol solutions (30, 50, 70, and 90%, and three changes of 100% for 10 min each), infiltrated, and embedded in Araldite resin. Ultra-thin sections (60 nm) were cut with a glass ultramicrotome (Leica Ultra cut UCT-GA-D/E-1/100) and placed on copper grids after incubation at 80°C for 72 h for complete polymerization. The sections were stained with uranyl acetate and counterstained with Reynolds lead citrate, with some alterations (Bozzola and Russell, 1998). A Hitachi 7500 (Japan) transmission electron microscope was used to take the images.

### Localization of ATG8 From Immunofluorescence Microscopy

For immunofluorescence, cells (3 × 10^6^ cells/ml) were fixed in a solution of 4% paraformaldehyde and 15% sucrose dissolved in phosphate saline buffer (PBS) for 1 h at RT Later, the cells were washed twice with PBS buffer. First, cells were permeabilized by incubation in 0.01% Triton X100 in PBS for 5 min at RT and washed twice in PBS. Next, the samples were transferred to sterile Eppendorf tubes and blocked with a 1% BSA (w/v) in PBS for 1 h. Samples were incubated with anti- ATG8 diluted (1:1000) in PBS buffer, pH = 7.2 containing 1% BSA overnight at 4°C on a rotatory shaker. Cells were then washed twice with PBS for 10 min at 25°C, followed by incubation in a 1:10000 dilution of the fluorescein Dylight 405 labeled goat anti-rabbit secondary antibody (Sigma) in PBS-BSA for 2 h at 25°C. Finally, cells were washed three times with PBS for 5 min. Images were captured with Carl Zeiss NL0 710 Confocal microscope. ATG8 localization was detected with excitation of Dylight 405 nm and emission at 420 nm by analyzing the images with ZEN software.

### Lipid Body Analysis by Nile Red Staining

We studied the impact of high light on non-polar lipid accumulation with confocal microscopy. Cells (3 × 10^6^ cells/ml) were collected after the 3rd-day growth in high light along with control. To stop the mobility of the cells, 5 μL Iodine solution [0.25 g in ethanol (95%)] was mixed and kept for 5 min under dark at RT. The cell suspension was then stained with Nile Red (NR, 1 μg mL^–1^ final concentration, Sigma, Sigma-Aldrich) followed by 20 min incubation in the dark. After staining, the samples were placed on a glass slide with a coverslip. Images were captured using Carl Zeiss NL0 710 confocal microscope. A 488 nm scanning laser was used with a 560–615 nm filter to detect neutral lipids. Samples were viewed with a 60× oil immersion lens objective, and the ZEN, 2010 software package was used for image analysis.

### Nile Red Visible Light Assay for Triacylglycerol

The *C. reinhardtii* cultures were diluted to a density of 3.0 × 10^6^ cells mL^–1^ and placed within 96 well microplate wells. To every well, a 5 μL aliquot of 50 μg mL^–1^ NR; (Sigma; ready in acetone) was added in 200 μl of equal density cells (3.0 × 10^6^ cells mL^–1^), and after a thorough mix, the plate was incubated in the dark for 20 min. The neutral lipids were then measured by visible light at a 485-nm excitation filter and a 595-nm emission filter employing a plate reader (Tecan infinite M200, Magellan). Quantification was achieved by using a standard curve ready with the lipid Triolein (Sigma T4792).

### Flow Cytometry Measurements

Flow cytometer Calibur (BD Falcon, United States) was use to quantify neutral lipid stain with NileRed. Cells were stained with 1 μg mL^–1^ NR (100 μg mL^–1^ stock in acetone) in the dark for 30 min before flow cytometry analysis. Measurement of 10,000 cells per sample was analyzed without gating and auto-fluorescence was nullified before measuring the NR fluorescence. The fluorescence reading was obtained at a 488-nm excitation filter and a 545-nm emission. The distribution of the cells stained with NR was expressed as a percent of the total.

### Total Pigment and Carbohydrate Quantification

*C. reinhardtii* cells were harvested by centrifugation (1,000 × *g* for 10 min) after the 3rd day at a cell density of 3.0 × 10^6^. Cell pellets were resuspended in methanol for chlorophyll extractions. The supernatant was used to measure the total chlorophyll (Porra et al., 1989). To calculate total carotenoid cell pellets were resuspended in 80% acetone, and the supernatant was used to measure the total carotenoid. Absorbance was measured at wavelength 480, 663, and 645 nm. Total carotenoid was calculated as = A480 + 0.114 (A663) − 0.638(A645) (Wellburn, 1952). Total carbohydrate was quantified by an Anthrone method (plate reader assay). For quantification of carbohydrate, the cell pellet was resuspended in 50 μL Milli-Q water and 150 μL of Anthrone chemical agent (0.1 g in 100 mL of conc. H_2_SO_4_ 98%) was added to the 50 μL algal cells. Initially, plates were incubated at 4°C for 10 min and then incubated at 100°C for 20 min. Later on, plates were placed at RT until color development. Plates were measured at 620 nm on an assay plate reader (Tecan M250), and total carbohydrate was calculated (Wase et al., 2019).

## Lipid Analysis and Quantification

For lipid extraction, 5-mg of lyophilized dry biomass was weighed equally from all the conditions. Total lipids were extracted as described by Bligh and Dyer’s method (Bligh and Dyer, 1959). Cell powder was resuspended in a mixture of methyl alcohol, chloroform (2:1), and 0.9% KCl. It was vortexed for 5 min and centrifuged at 3,000 × *g* for 5 min for phase separation. The organic layer (lower layer phase) was transferred to a clean Eppendorf tube. The solvent was evaporated by purging nitrogen gas. The dried extract was then dissolved in 50 μL chloroform. For standard, TAG (Sigma-Aldrich, St. Louis, United States) was dissolved in CHCl_3_ (1 mg mL^–1^). Around 0.5 μg of lipid extract was loaded as a spot on 20 cm × 20 cm silica gel TLC plates. For neutral lipids separation, the TLC plate was developed in a mixture of hexane/diethyl ether/acetic acid (17:3:0.2), and polar lipids (membrane lipids) were developed in a mixture of acetone/toluene/water (91:30:8) according to Wang and Benning (2011). Bands were visualized by staining with iodine vapor and identified the membrane lipids as cited above. For quantitative analysis, individual lipids were extracted from the TLC plates with a razor blade transferred into a glass tube followed by *trans* methylation to fatty acid methyl esters (FAMEs). FAME of the samples were identify quantitatively by GC (gas chromatographs) 6,890 fitted with 25 m × 0.2 mm phenylmethyl silicone fused silica capillary. The temperature program ramps from 170 to 270°C at 5°C per min. Following the analysis, a ballistic increase to 300°C allows cleaning of the column during a hold of 2 min. The amounts of fatty acids were calculated based on the content of fatty acids derived from GC using heptadecanoic acid (Sigma-Aldrich, St. Louis, United States) as an internal standard.

### Fatty Acid Methyl Esters Analysis for the Total Lipids

The 5 mg lyophilized sample was dissolved in 1,000 μL of acetonitrile (ACN), 0.1% formic acid (FA) (v/v), and vortexed for 1 h at 900 rpm at RT. The solution was then centrifuged at 13,000 rpm for 10 min at 4°C. 100 μL of supernatant was collected, and the pellet was dried using a speed vacuum. The pellet was dissolved in 90 μL of acetonitrile 0.1% formic acid (v/v) and spiked with 10 μL of the standard internal mixture (Table 1). The solution was vortexed for 30 min at 750 rpm at RT and speed vacuumed to dry at 40°C. The extracted lipids were derivatized with *n*-Butanol for 20 min at 60°C, then again speed vacuumed to dry at 60°C for 15 min. The dried pellets were dissolved in 100 μL of ACN 0.1% FA and vortexed for 5 min at 750 rpm on a thermomixer. The samples were then centrifuged at 13,000 rpm for 10 min at RT. The supernatant was taken into HPLC sample vials for further analysis. The 100 μL extracted lipid samples were brought to RT and filled into individual HPLC vials with 200 μL inserts and placed into the autosampler of Nexera X2 UFLC, connected to LC-MS/MS (SHIMADZU 8045) MS system with an ESI source. The data was analyzed by Lab solutions software, and the concentrations were calculated by measuring the area under the curve (AUC) for the internal standards.

**TABLE 1:** Fatty acid content of *C.reinhardtii* under normal and high light conditions.

Fatty acid	WT (50 μmol photons m^–2^ s^–1^) % ± *S.D*	WT (500 μmol photons m^–2^ s^–1^) % ± *S.D*	*pgrl1* (50 μmol photons m^–2^ s^–1^) % ± *S.D*	*pgrl1* (500 μmol photons m^–2^ s^–1^) % ± *S.D*	*pgr5* (50 μmol photons m^–2^ s^–1^) % ± *S.D*	*pgr5* (500 μmol photons m^–2^ s^–1^) % ± *S.D*
C14:0	0.237 ± 0.003	0.546 ± 0.004	0.185 ± 0.001	0.176 ± 0.001	0.594 ± 0.003	0.609 ± 0.003
C14:1	0.182 ± 0.002	0.589 ± 0.005	0.42 3 ± 0.002	0.154 ± 0.001	0.329 ± 0.002	0.508 ± 0.003
C14:2	0.296 ± 0.003	0.767 ± 0.003	0.423 ± 0.002	0.132 ± 0.001	0.632 ± 0.003	0.406 ± 0.002
C14:3	0.123 ± 0.002	0.412 ± 0.003	0.318 ± 0.002	0.64 ± 0.003	0.228 ± 0.001	0.216 ± 0.001
C16:0	1.182 ± 0.002	1.412 ± 0.003	2.123 ± 0.007	3.415 ± 0.007	3.45 ± 0.005	7.132 ± 0.006
C16:1	1.070 ± 0.006	2.391 ± 0.021	1.874 ± 0.004	4.178 ± 0.006	2.212 ± 0.003	4.398 ± 0.001
C16:2	0.192 ± 0.002	1.764 ± 0.004	0.36 ± 0.002	2.544 ± 0.003	0.573 ± 0.003	5.027 ± 0.005
C16:3	1.213 ± 0.019	4.76 ± 0.008	1.816 ± 0.009	2.78 ± 0.014	2.1 ± 0.010	3.13 ± 0.016
C18:0	0.499 ± 0.002	1.12 ± 0.004	1.659 ± 0.008	2.061 ± 0.012	1.025 ± 0.005	3.752 ± 0.007
C18:1	0.168 ± 0.001	0.942 ± 0.003	0.439 ± 0.002	1.998 ± 0.004	0.826 ± 0.004	5.46 ± 0.012
C18:2	0.427 ± 0.002	0.200 ± 0.001	0.651 ± 0.003	0.212 ± 0.001	0.199 ± 0.003	1.15 ± 0.003
C18:3	3.124 ± 0.018	7.200 ± 0.029	2.725 ± 0.029	5.895 ± 0.014	5.738 ± 0.109	7.266 ± 0.046
C18:4	1.21 ± 0.005	2.56 ± 0.019	2.114 ± 0.011	1.23 ± 0.006	3.161 ± 0.04	3.395 ± 0.017
ΣSFA	1.918 ± 0.016	3.078 ± 0.007	3.967 ± 0.016	5.652 ± 0.02	5.069 ± 0.02	11.493 ± 0.016
ΣMUFA	1.42 ± 0.007	3.92 ± 0.008	2.736 ± 0.027	5.33 ± 0.019	3.367 ± 0.011	10.36 ± 0.025
ΣPUFA	4.192 ± 0.051	17.46 ± 0.059	8.407 ± 0.203	12.43 ± 0.072	12.631 ± 0.042	20.59 ± 0.09
ΣTFA	7.53 ± 0.079	24.45 ± 0.091	15.11 ± 0.252	25.415 ± 0.101	21.067 ± 0.073	42.449 ± 0.131

*The data represents as % of total fatty acid for n = 3. SFA, saturated fatty acids; MUFA, monounsaturated fatty acids; PUFA, polyunsaturated fatty acids; TFA, total fatty acids.*

### Immunoblots

Cells were collected by centrifugation. The cell pellet was solubilized in protein extraction buffer (0.1 M DDT, 4% SDS, and 0.1 M Tris, pH- 7.6), and all the samples were kept for heating at 95°C for 5 min. Cell suspensions were centrifuged at 2,810 × *g* for 10 min, and the supernatant was collected. Protein concentration was quantified using the Bradford method, and 5 μg of proteins were loaded per well. The individual proteins were separated with 15% Bis-Tris gels for diacylglycerol acyltransferases (DGAT2A) and PDAT1 proteins and 12% for ATG8 and Histone3 (H3) proteins. Separated proteins were then transferred to a nitrocellulose membrane (Towbin et al., 1979). Specific primary antibodies were purchased from Agrisera (Sweden) and used at dilutions of DGAT2A (1:2500), PDAT (1:2500), and ATG8 (1:2500). H3 (1:10,000) was used to see equal loading. The secondary antibody, anti-IgG raised from Goat (1:10,000), was used for detection. Immunoblots were obtained by the Chemi-Doc touch imaging system (Bio-Rad) by using Chemiluminescence.

### Statistical Analysis

All experiments were conducted in triplicate, and data are obtained as the mean ± standard deviation (SD). Statistical analysis was done using Sigma plot 14.5 software. One-way ANOVA and subsequent Tukey Test were used to analyze the statistical significance (*p < 0.05, ^**^p < 0.01, ^***^p < 0.001) of the data.

## Results

### Growth and Pigment Analysis Under High Light

**FIGURE 1:**
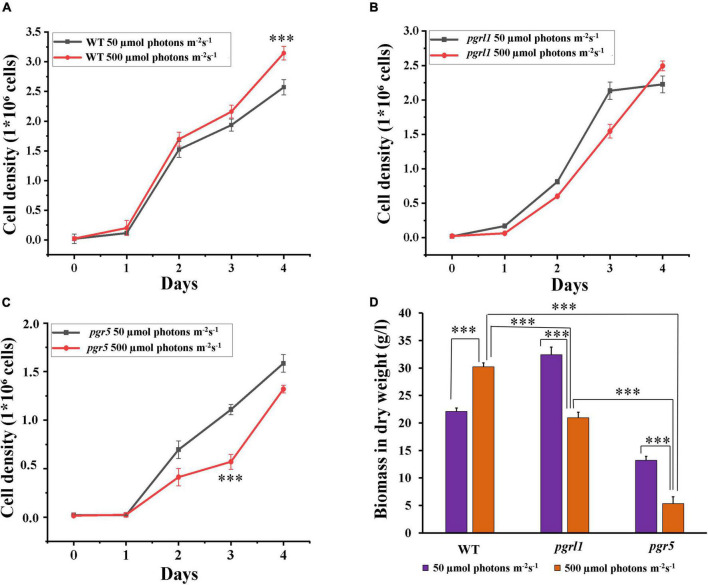
Cell growth and dry biomass were monitored from cells grown under normal light and high light conditions. **(A–C)** Cell growth was analyzed by cell counting by hemocytometer collected for every 24 h of WT (CC124), **(B)**, proton gradient regulation 1 (*pgrl1*), and **(C)**, proton gradient regulation 5 (*pgr5*) from 50 μmol^–2^ s^–1^ and 500 μmol photons m^–2^ s^–1^ conditions. **(D)** Total biomass in dry weight was calculated after the 3rd day of growth of cells (WT, *pgrl1*, and, *pgr5*) grown from light conditions. All the experiments were done three independent times, and error bars represent the mean ± SD (*n* = 3). Statistical comparison was performed using one- way analysis of variance (ANOVA) followed by the Tukey multiple comparison tests, and *p*-values obtained are indicated asterisks (^***^p < 0.001).

The WT, proton gradient regulation (*pgrl*)*1* and *pgr5* (cyclic electron transport dependant mutants) were grown in Tris-acetate phosphate (TAP) medium at light intensities of 50 (as a control) and 500 μmol photon m^–2^ s^–1^ for 4 days. Under 50 μmol photon m^–2^ s^–1^ light intensity, all the cultures grew normally (Figure 1). The growth of WT cultures increased (Figure 1A), but *pgrl1* and *prg5* mutants exhibited slower growth in high light conditions after 2 days (Figures 1A–C). Our results showed that the cell growth pattern of the WT was increased 30%, whereas *pgrl1* and *pgr5* growth were decreased 21 and 30% compared to the WT under high light (500 μmol photon m^–2^ s^–1^) after 3rd day and normalized OD data was shown in Supplementary Table 1. Mainly, the *pgr5* mutant shows impaired growth, whereas *pgrl1* was not affected under the same high light condition (500 μmol photon m^–2^ s^–1^) after 3rd day of growth (Figures 1B,C). We have studied the effect of light intensity on biomass. The WT shows an increase in biomass yield 75.5% in high light, however, it reduced 64.6 and 43.6% in *pgrl1* and *pgr5*, respectively, after the 3rd day (Figure 1D). However, after the 4th day, biomass is almost the same in both mutants when grown in high light.

**FIGURE 2:**
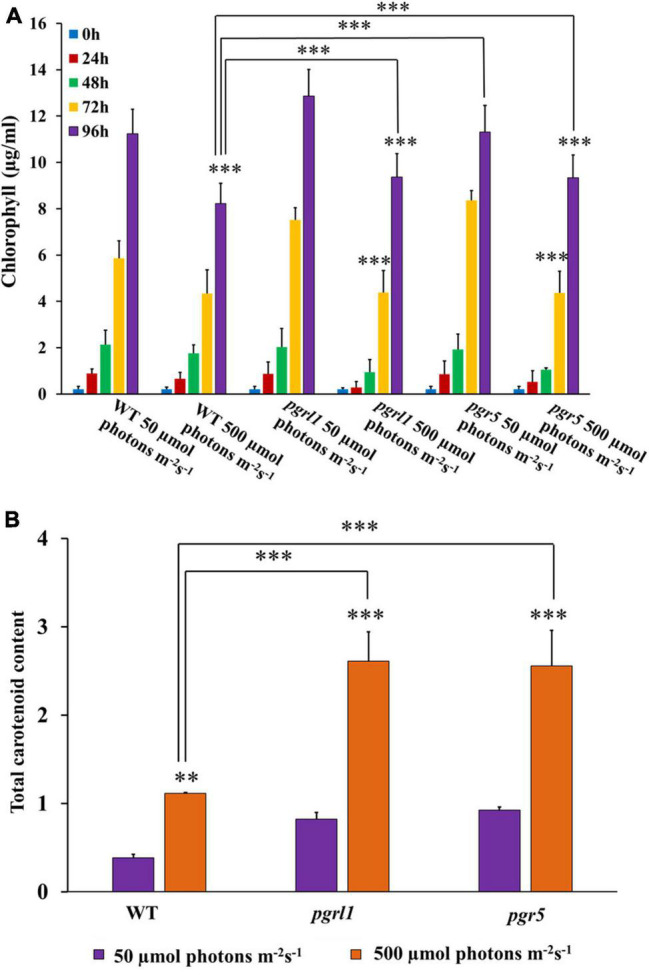
Chlorophyll and carotenoid content were quantified from cells grown under normal and high light conditions. **(A)** Chlorophyll content was quantified from 50 μmol photons m^–2^s^–1^ and 500 μmol photons m^–2^ s^–1^ conditions with different time intervals of 24, 48,72, and 96 h. **(B)** Total carotenoid content from the cells grown under normal and high light conditions. Carotenoids were calculated from cells grown with control WT and mutant *pgrl1* and *pgr5* cells grown from normal light (50 μmol photons m^–2^ S^–1^) and high light condition (500 μmol photons m^–2^ S^–1^) measured after 3rd day. Three biological experiments were done, *n* = 3. Statistical significance was analyzed using one-way ANOVA with Tukey test and *p*-value indicated as (^***^p < 0.001, ^**^p < 0.01).

To determine the effect of high light on the pigment level associated with *C. reinhardtii* photosynthetic complex, we have measured the chlorophyll and carotenoid content from standard and high light-grown cells. Chlorophyll content was significantly reduced in *pgrl1* and *pgr5* mutants, respectively, under high light intensity (Figure 2A). At the same time, we observed twofold increase in carotenoid content in *pgrl1* and *pgr5* under high light (Figure 2B). It is known that increased carotenoid content acts as a protective mechanism against light stress in the *C. reinhardtii* (Li et al., 2009). Carotenoids under high light conditions generally tend to increase to protect PSII from photoinhibition. Data shows that an increase in light intensity results in decreased specific growth rate, increased carotenoid, and reduced biomass production of *pgrl1* and *pgr5*. We assume that reduced growth might be due to ROS production. Our results have elicited us to check further studies and detect the ROS in high light stress.

### Reactive Oxygen Species Induction in High Light

**FIGURE 3:**
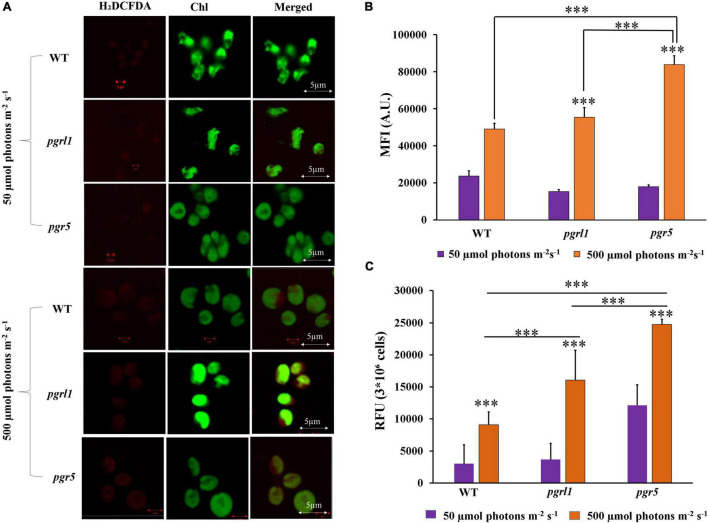
Total Reactive oxygen species (ROS) were measured with H_2_DCFDA. **(A)** *C. reinhardtii* cells of WT, *pgrl1* and *pgr5* were collected from the mid-log phase grown under normal (50 μmol photons m^–2^ S^–1^) and high light (500 μmol photons m^–2^ S^–1^) for 3rd day. Measured ROS using 2,7dichlorodihydrofluorescein diacetate (H_2_DCFDA) (10 μM) staining, and cells were imaged using the Zeiss 510 confocal microscope. The images were collected with three individual measurements for all the conditions (*n* = 3) and analyzed with Zeiss software. Scale bars = 2 μm. **(B)** Quantification of the fluorescence intensity done by ImageJ. **(C)** Total ROS was also quantified by spectrophotometry of WT, *pgrl1* and *pgr5* grown under light condition of 3rd day. Three individual measurements were performed for all the conditions (*n* = 3). Statistical significance was analyzed using one-way ANOVA with Tukey test and the *p*-value obtained are indicated (^***^p < 0.001).

To determine the oxidative stress, cells were stained with 2′,7′-dichlorodihydrofluorescein diacetate (H_2_DCFDA). ROS was observed using confocal microscopy as well as quantified by spectrophotometry (Figures 3A,C), where an increase in ROS was observed under the high light condition in WT, *prgl1*, and *pgr5* (Figure 3). However, this increase was predominant in *pgr5* (Figures 3A–C). The generation of more ROS in *pgr5* could probably explain the reduced growth rate. Further, these results suggest that ROS formation under high light may contribute to autophagy and lipid accumulation. Reports show that ROS may induce multiple metabolic functions like lipid metabolism and autophagy (Shi et al., 2017). High light is one of the factors responsible for ROS formation because of over reduction of the photosynthetic electron transport chain, which might generate ROS that induces autophagy in *C. reinhardtii* (Pérez-Pérez et al., 2012). Based on the above reports, we have conducted experiments related to autophagy caused by high light.

### Cellular Localization of ATG8

**FIGURE 4:**
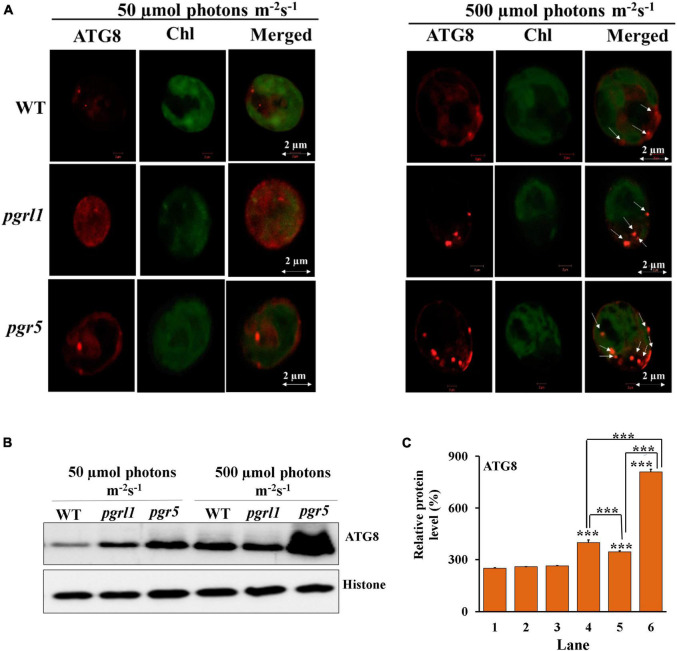
Autophagy (ATG)8 accumulation in *C. reinhardtii* cells under high light conditions. **(A)** Autophagy induction in *C. reinhardtii* cells collected at phase cells (3.0 × 10^6^ cells) grown in tris-acetate phosphate (TAP) medium under continuous light of 50 and 500 μmol photons m^–2^ s^–1^. Samples were collected after 3rd day and immunoassay with anti-ATG8 antibody (in red). Chloroplasts were visualized by auto-fluorescence (in green). The images were collected with three individual measurements for all the conditions (*n* = 3). The image was taken using a Zeiss confocal microscope. Scale bars = 2 μm. **(B)** Protein identification by western blot. Western blot analysis of ATG8 evaluated from 3rd-day cells under the normal (50 μmol photons m^–2^ s^–1^) and high (500 μmol photons m^–2^ s^–1^) light condition, and each lane 5 μg protein were loaded. Autophagy (ATG)8-PE protein was resolved on 15% Bis-Tris gel in denaturing conditions. Histone (H3) was used as a loading control. All the blots were conducted in three independent experiments (*n* = 3) and obtained similar results. **(C)** Quantification of the immunoblots was done by ImageJ. The measurements were repeated with three individual cultures, and error bars represented the mean ± SD (*n* = 3). Statistical significance was analyzed using one-way ANOVA with Tukey test and the *p*-value obtained are indicated (***p < 0.001).

We have used an anti -ATG8 antibody to examine this protein’s cellular localization in immunofluorescence microscopy (Figure 4A). Autophagy (ATG)8 proteins localize as small red dots in the cytoplasm under the normal light condition in WT, *pgrl1*, and *pgr5* mutant. However, under high light conditions, number of spot significantly increased in *pgr5* (40%) (Supplementary Figure 1), which shows activation of autophagy and formation of autophagosomes in close agreement with high accumulation of ATG8 [phosphatidylethanolamine (PE) modified ATG8] detected by western blotting. The autophagy-related protein ATG8-PE was significantly expressed in *pgr5* in high light conditions (Figures 4B,C). Upon autophagy activation, ATG8 binds to the autophagosome membrane through phospholipid phosphatidylethanolamine (PE) to form ATG8-PE in a process called lipidation. Hence autophagy may play a significant role in the accumulation of lipids in high light conditions in *pgr5*. Therefore, in line with autophagy, the accumulation of lipids is lower in WT and *pgrl1*. However, the ATG8 expression is predominant in *pgr5* under high light conditions. These results suggested that induction of ROS due to high light may induce autophagy in *pgr5* than WT and *pgrl1* (Pérez-Pérez et al., 2012; Perez-Martin et al., 2014). Therefore, it may trigger the lipid metabolism in *C. reinhardtii* cells.

### Lipid Droplet Analysis From Confocal Microscopy

**FIGURE 5:**
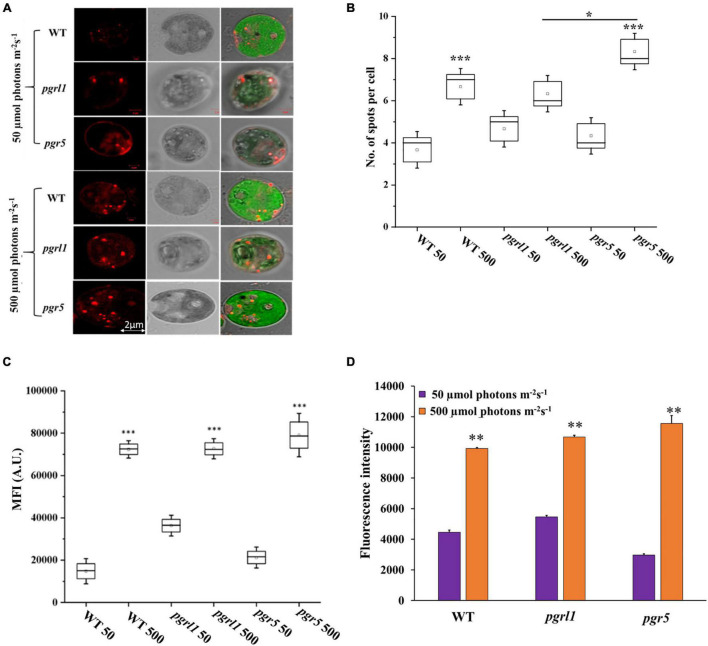
Lipid droplets were identified through confocal microscopy and FACS analysis. **(A)** Cells grown under normal (50 μmol photons m^–2^ s^–1^) and high (500 μmol photons m^–2^ s^–1^) light conditions were stained with Nile Red (5 μM/mL) for lipid droplets in *C. reinhardtii* strains, WT, *pgrl1*, and *pgr5*. The images were collected with three individual measurements for all the conditions. Bars = 2 μm. **(B)** Quantification of number (quantification was performed from single cell with three individual cultures) and **(C)** fluorescence intensity of lipid droplets as shown. Box plots indicate the medians, means, and quartiles. Statistical significance was analyzed using one-way ANOVA with Tukey test, and the *p*-value obtained is indicated (^***^p < 0.001, *p < 0.05). Bars = 2 μm. **(D)** Fluorescent activated cell sorter (FACS) analysis was carried out for lipid accumulation with BD Fortessa, United States. WT, *pgrl1* and *pgr5* 50; WT, *pgrl1* and *pgr5* 500, represents 50 and 500 μmol photons m^−2^ s^−1^. All the measurements were done with the PE-A filter. The analysis showed mean fluorescence value to compare light conditions of normal (50 μmol photons m^–2^ s^–1^) and high (500 μmol photons m^–2^ s^–1^). An unstained condition was kept to minimize the chlorophyll autofluorescence from cells. Cells were stained with Nile Red (NR), and measurements were done after incubated for 20 min under dark at room temperature (RT). The measurements were repeated with three individual cultures, and error bars represented the mean ± SD (*n* = 3). Statistical significance was analyzed using one-way ANOVA with Tukey test and *p*-value obtained are indicated (^***^p < 0.001, ^**^p < 0.01, *p < 0.05). Bars = 2 μm.

We have stained the cells with Nile red (NR) for lipid studies, binds explicitly to lipid bodies in the cell, and gives a characteristic yellow-orange fluorescence. Our results clearly show the overall increase of lipid droplets under high light in WT, *pgrl1*, and *pgr5* mutants (Figures 5A,B and Supplementary Figure 2). When we measure the fluorescence intensity from the confocal images, the number of lipid spots is higher in high light-grown cells, but it is more significant in *pgr5* (Figure 5C). However, autofluorescence due to chlorophyll was less in *pgrl1* and *pgr5* than in control, indicating decreased chlorophyll content under high light, even though cells had a significant accumulation of lipid bodies (Figure 5A). Lipid accumulation was observed in all strains under high light conditions (Goold et al., 2016). However, lipid accumulation is more significant in *pgr5* (60%) than other strains under high light. We have tested NR fluorescence from fluorescent activated cell sorter (FACS) to assess the lipid accumulation in high light conditions. We obtained twofold higher mean fluorescence intensity from all the strains with high light than normal growth conditions (Figure 5D).

### Neutral Lipid Identification by Nile Red Fluorescence

The neutral lipids were semi-quantified by using NR fluorescence from an equal number of cells. NR fluorescence results show that the neutral lipid level was increased with equal cell density. NR fluorescence of *C. reinhardtii* cells WT, *pgrl1*, and *pgr5* were calculated every 24 h while growing them in light at 50 and 500 μmol photon m^–2^ s^–1^ (Supplementary Figure 3). After 24 h, cells did not cause any change in total lipid content. However, after 48 h of high light growth, there was an increase in NR fluorescence.

In contrast, there is an increase in signal at 750 nm. Overall, high light-grown cells exhibit a relative two-fold increase in neutral lipid content over 3–4 days. However, this accumulation of neutral lipids is predominantly more in the *pgr5* mutant when compared to other strains. Therefore, our results indicate that an increase in light intensity facilitates the production of neutral lipids in *C. reinhardtii*. Further, we have performed electron microscopic studies to determine whether high light induces lipid accumulation in the cells.

### Transmission Electron Microscopy Studies

**FIGURE 6:**
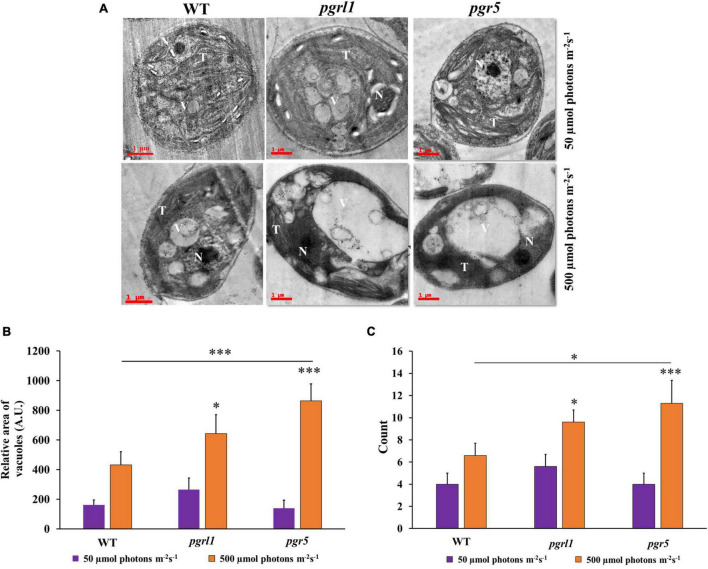
Ultrastructure analysis of *Chlamydomonas* cells during normal (50 μmol photons m^–2^ s^–1^) and high light (500 μmol photons m^–2^ s^–1^). **(A)** Transmission electron micrographs of *C. reinhardtii* cells WT and mutants *pgrl1* and *pgr5* grown under photoheterotrophic conditions under normal (50 μmol photons m^–2^ s^–1^) and high light (500 μmol photons m^–2^ s^–1^). Representative images for the cells sampled after the 3rd day, N-nucleus; O, T-thylakoid membranes; V-vacuole. Three independent experiments were conducted from each sample (*n* = 3). Scale bar = 1 μm. **(B)** Quantification of area vacuoles. **(C)** The number of vacuoles. Quantification and number of vacuoles were performed from single cell with three independent cultures. Statistical significance was analyzed using one-way ANOVA with Tukey test and the *p*-value obtained are indicated (^***^p < 0.001, *p < 0.05).

To see the clear lipid vacuoles in the cell, we have carried out transmission electron microscopy (TEM). It reveals that *pgrl1* and *pgr5* exhibit a large cytoplasmic vesicle under high light-grown cells (Figures 6A–C and Supplementary Figure 4). It showed that the membrane structure is aberrant under high light, especially the *pgrl1* and *pgr5* mutants. The majority of the thylakoid membranes were loosen in high light-grown cells. While in the WT cells, the thylakoid membranes are arranged as layers (stacked) in the chloroplast, with some membrane appressed. Recently we reported that photosynthetic efficiency is reduced under the high light condition in WT, *pgrl1*, and *pgr5* mutants, which may be due to a change in chloroplast structure (Yadav et al., 2020). Such phenotype raised the question that mutant strains are defective in generating the functional membrane under high light conditions. The earlier report emphasized that autophagic bodies accumulate in plant cell vacuoles under Concanamycin A treatment since vacuolar hydrolase cannot act (Thompson et al., 2005). However, in our case, under high light conditions, we have observed a high degree of vacuolization and an increase of vacuole size in mutants (Figures 6A,B and Supplementary Figure 4). It was also being reported that vacuole lytic function is needed to synthesize TAG and lipid bodies in *C. reinhardtii* cells when subjected to light stress (Couso et al., 2018).

### Carbohydrate Assay

**FIGURE 7:**
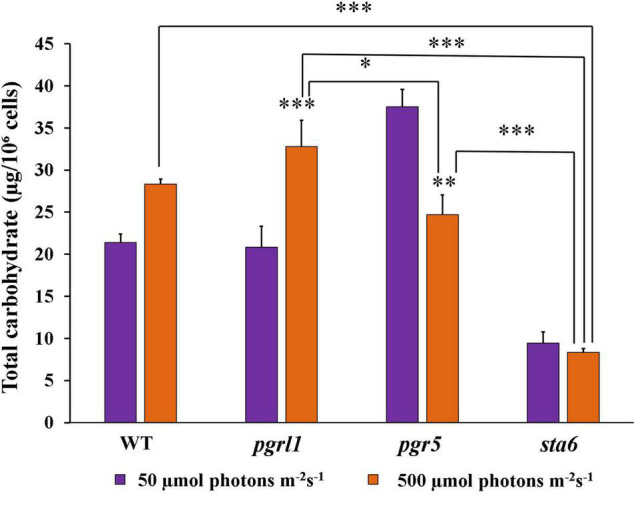
Total carbohydrate content quantified by Anthrone method from the cells grown under normal to high light conditions. Cells were collected at the mid-log phase from normal (50 μmol photons m^–2^ s^–1^) and high (500 μmol photons m^–2^ s^–1^) light conditions. The color was measured with spectrophotometry at 620 nm. Three biological experiments were done (*n* = 3), and error bars represent the mean ± SD (*n* = 4). Statistical significance was analyzed using one-way ANOVA with Tukey test and *p*-value obtained are indicated (^***^p < 0.001, ^**^p < 0.01, *p < 0.05).

Carbohydrate is a polysaccharide, and it is the storage form of sugars and starch in plants/algae. We quantified the carbohydrate content in WT, *pgrl1*, and *pgr5* strains in normal and high light conditions. The carbohydrate content of WT and *pgrl1* was increased significantly under the high light condition. The carbohydrate content was cross-checked with a starch deficient mutant (*sta6*) under normal light and high light. It shows about 8 μg of carbohydrate/10^6^ cells in standard and high light conditions (Figure 7) as measured with the anthrone reagent, which estimates starch and soluble sugars. Overall, our reports indicate that carbohydrate content has increased under the high light condition in WT and *pgrl1*. However, it reduced in *pgr5* mutant under high light. Microalgae change their metabolism and convert excess energy into an energy-rich product such as lipid, starch, carbohydrate, or proteins under unfavorable conditions (Heredia-Arroyo et al., 2010). However, *pgr5* may have an altered NADPH and ATP ratio during photosynthesis, switching metabolic pathways that lead to lipid accumulation. These results indicate that high light induces the lipid pathways due to more energy equivalents in the *pgr*5 mutant. Further, we have tested the protein/enzyme of the lipid pathway from all the conditions.

### Protein Analysis by Immunoblot

**FIGURE 8:**
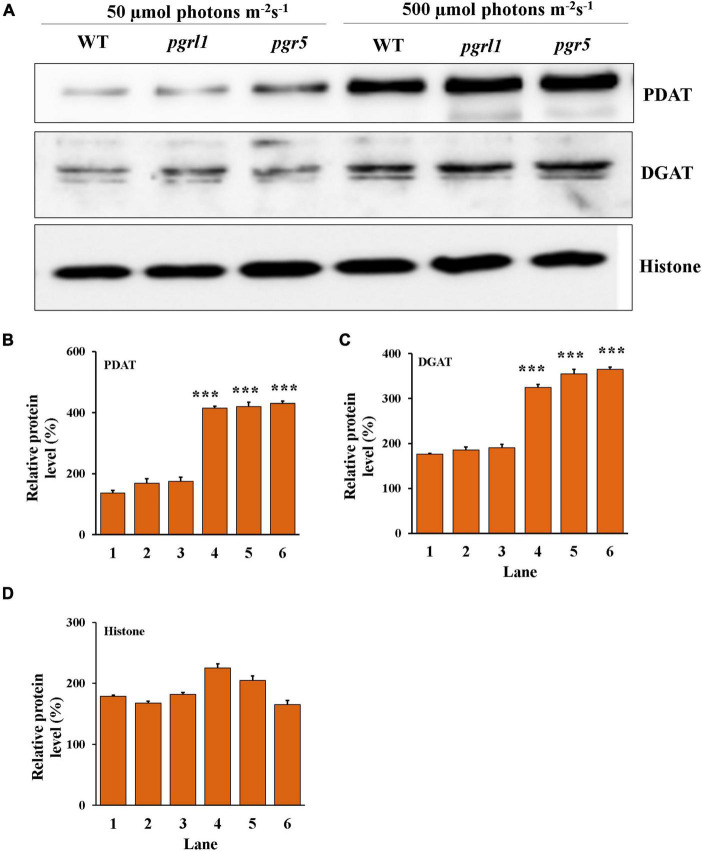
Protein identification from western blot analysis. **(A)** The proteins were separated by denaturing gel electrophoresis, transferred to nitrocellulose membranes, and probed for the indicated proteins. Western blot analysis of Acyl-CoA: diacylglycerol acyltransferase (DGAT2A) and phospholipid diacylglycerol acyl-transference (PDAT1) evaluated from 3rd-day cells under the normal (50 μmol photons m^–2^ s^–1^) and high (500 μmol photons m^–2^ s^–1^) light condition, and each lane (5 μg) protein were loaded on to 10% Bis-Tris gel in denaturing condition. Histone (H3) was used as a loading control. All the blots were in three independent times (*n* = 3) and obtained similar results. **(B)** Quantification of the immunoblots of PDAT, **(C)** DGAT and **(D)** histone. Data are expressed as mean ± SD of 3 replicates. Statistical significance was analyzed using one-way ANOVA with Tukey test and *p*-value obtained are indicated (***p < 0.001).

We have focused on the enzymes involved in the triacylglycerol (TAG) synthesis known as the Kennedy pathway. This pathway is mainly catalyzed by the acylation of diacylglycerols (DAGs) by diacylglycerol acyltransferases (DGAT), in which acyl-CoA is a substrate to form TAG. Acylation of diacylglycerols (DAGs) by phospholipid: diacylglycerol acyltransferase 1 (PDAT1) enzyme is the acyl-CoA independent pathway in the ER. To determine the involvement of PDAT and DGAT in high light response in *C. reinhardtii* and their protein level regulation was calculated (Figures 8A–C). The PDAT and DGAT were transiently upregulated in response to high light conditions. Protein expression was increased after the 3rd day, specifically in *pgr5* expression of PDAT compared to *pgrl1* (Figure 8A). Our results confirm an increase in TAG in *pgrl1* and *pgr5* due to the enhanced content of PDAT and DGAT enzymes.

### Fatty Acid Composition Under High Light Condition

Liquid chromatography/mass spectrometry (LC/MS) was used to analyze the fatty acids transesterification and the resultant fatty acid methyl esters (FAMEs) from control strains WT, and mutants *pgrl1* and *pgr5* under normal (50 μmol photons m^–2^ s^–1^) and high light (500 μmol photons m^–2^ s^–1^) cells (Table 1). Different compositions of fatty acids are observed in the high light grown *C. reinhardtii.* There was an increase in C16:1, C16:2, C16:3, C18:0, C18:1, and C18:3 in *pgrl1* and *pgr5* mutant under high light conditions. We recently observed increasing total fatty acids in severe Fe deficiency from *C. reinhardtii* (Devadasu et al., 2019; Devadasu and Subramanyam, 2021). In our case also the total fatty acid content is significantly increased in *pgrl1* and *pgr5* mutant strains. Overall saturated fatty acid increases from 6 to 10%, 13 to 18%, and 12 to 37% in WT, *pgrl1*, and *pgr5* mutant, respectively, and monounsaturated fatty acid increases from 5 to 15%, 10 to 20%, and 12% to 38% in WT, *pgrl1*, and *pgr5* mutant, respectively. However, there are also changes in total polyunsaturated fatty acids from 6 to 23%, 11 to 16%, and 17 to 27%. This result implies that high light increases SFA and MUFA in WT, *pgrl1*, and *pgr5* mutant strains, but specifically, the SFA and MUFA increased significantly *in pgr5*.

### Neutral Lipid and Membrane Lipid Analysis

**FIGURE 9:**
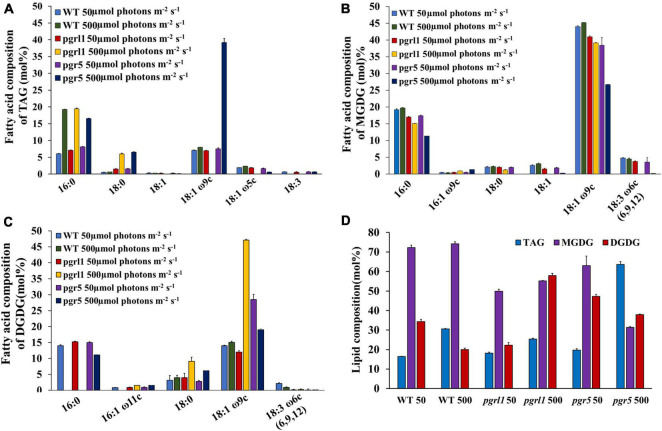
Changes in lipid composition of *C. reinhardtii* cells grown under high light conditions. **(A)** Fatty acid composition of the TAG of WT, *pgrl1*, and *pgr5* under normal (50 μmol photons m^–2^ s^–1^) and high light (500 μmol photons m^–2^ s^–1^). Fatty acids are represented as the total carbon number followed by the number of double bonds. The position of specific double bonds is indicated from the methyl end ‘ω.’ **(B)** Fatty acid composition of MGDG. **(C)** Fatty acid composition of DGDG. **(D)** Changes in major lipid class in WT, *pgrl1*, and *pgr5* under high light (500 μmol photons m^–2^ s^–1^). In the ‘x’ axis of **(D)**, WT, *pgrl1* and *pgr5* 50, WT, *pgrl1* and *pgr5* 500, represents 50 and 500 μmol photons m^–2^ s^–1^. Averages from two independent experiments and their standard deviations are shown. TAG, triacylglycerol; PG, phosphoglyceride; DGTS, diacylglycerol-trimethyl homoserine; MGDG, monogalactosyldiacylglycerol; and DGDG, digalactosyldiacylglycerol. Averages from two replicate experiments and their standard deviations are shown.

To examine the fatty acid formed under high light, we studied the changes in neutral lipid, especially TAG and membrane lipid content and composition. We prepared neutral lipid extract from equal dry weights of WT, *pgrl1*, and *pgr5* cells under 50 and 500 μmol photon m^–2^ s^–1^. Further, the extracted lipids were separated on TLC plates from the 3rd day of culture. This was stained with iodine, and results revealed that accumulation of more TAG was observed in *pgr5* compared to *pgrl1* and WT (Supplementary Figure 5). To quantify the TAG, the TLC bands were recovered, and FAMES quantifies TAG content through of GC (Figure 9). TAG concentration in WT and *pgrl1* increased from 9 to 18%; however, *pgr5* significantly increased from 11 to 34% (Figure 9D). The fatty acid composition of TAG bands, 16:0, was raised in all strains under high light. However, the level of 18:0 was increased significantly in *pgrl1* and *pgr5* mutants. Further, 18:1ω9c majorly increased in *pgr5* mutant under high light (Figures 9A,D). The accumulation of a significant amount of TAG in *pgrl1* and *pgr5* under high light may suggest the role of membrane lipid in TAG synthesis (Fan et al., 2011).

To assess all the membrane lipids of *C. reinhardtii*, TLC is carried out. In WT, we observed not much change in chloroplast-specific lipid monogalactosyldiacylglycerol (MGDG), but chloroplast lipid digalactosyldiacylglycerol (DGDG) was marginally increased. However, in *pgr5* mutant, MGDG and DGDG content reduced significantly under high light conditions (Supplementary Figure 5). Further GC quantification confirmed that the MGDG and DGDG were significantly decreased in high light (Figures 9B,D). The fatty acid composition shows that DGDG 18:1 and 18:1ω9c increased in *pgrl1* mutant while decreased in *pgr5* mutant; however, not much change was observed in WT (Figures 9C,D). Further, the MGDG is enriched with C16:0, C18:1 ω9c, C18:3, and C18:0. Not much change was seen in the fatty acid composition of MGDG in WT and *pgrl1* but significantly decreased in *pgr5* under high light (Figures 9B,D). We assume that the reduced amounts of MGDG and DGDG are metabolized to TAG synthesis in *pgr5*, and a similar report was also observed from other studies (Li et al., 2008; Devadasu and Subramanyam, 2021). The TLC and the GC data of membrane lipids support that the electron microscopic data that the stacks of thylakoids disturbed because of change in membrane lipids. Thus fatty acids from the chloroplasts and other intracellular membrane systems may have converted into TAG.

## Discussion

Cyclic electron transport around PSI requires the functions of PGRL1 and PGR5 to generate a proton gradient over the thylakoid membrane. PGR5 plays a regulatory role in cyclic electron flow around the PSI. It indirectly protects the PSI by enhancing photosynthetic control, a pH-dependent down-regulation of electron transfer at the Cyt *b*6*f* (Buchert et al., 2020). Less proton motive force across the thylakoid membrane and reduced cyclic electron transport around PSI suggest these protein’s pivotal role in photosynthesis to protect it from high light (Yadav et al., 2020). Therefore, the CET is diminished in these mutants. It is known that CET plays a significant role in protecting plants in light stress. Recently, we reported that the photosynthetic activity was severely affected in *pgrl1* and *pgr5* of *C. reinhardtii* when the cells were grown in high light (Yadav et al., 2020). We have also observed the non-photochemical quenching is substantially reduced in *pgr5*, which is supposed to protect the algae from high light that could harmlessly dissipate excess excitation energy as heat (Nama et al., 2019). When they grow in photoautotrophic conditions, the *pgr5* cells were much more sensitive to high light (500 μmol photon m^–2^ s^–1^). To our knowledge, in *pgrl1* and *pgr5*, the autophagy-induced lipid accumulation has not been explored from high light conditions despite being well characterized on the photosynthesis. This study shows that autophagy induces lipid accumulation under high light in *pgrl1* and *pgr5* mutants.

### High Light Effect the Growth and Chlorophyll Content

Light is an essential factor in the production of biomass. Half of the microalgae species’ dry weight is carbon and lipid (Zhu, 2015). This study demonstrates that high light intensity (500 μmol photons m^–2^ s^–1^) enhanced the growth rate, promoted biomass and lipid accumulation of *C. reinhardtii* compared to regular light intensities in WT. However, growth and biomass were reduced in mutant (Figures 1B–D). PGRL1 and PGR5 proteins are essential in the acclimation process to high light. Thus, the growth curves show that the absence of these leads to less biomass and growth (Figure 1). In addition, the light stress response has been previously studied and shows the importance of light intensity in producing biomass and fatty acids in microalgae (Nzayisenga et al., 2020).

### High Light Induces the Reactive Oxygen Species and Autophagy in *C. reinhardtii*

On the other hand, when microalgal cells were grown in high light, PSII undergoes severe damage. Hence photosynthetic electron transport chain induces the ROS (Li et al., 2009). Antioxidants usually accumulate in cells like carotenoids to repair the damage caused by ROS (Perez-Martin et al., 2014). Similarly, we also observed in our results that the total carotenoid content increased (Figure 2B). Interestingly, the ROS generation was much higher in *pgr5* because the lack of this gene led to acceptor side limitations as PGR5 is involved in protecting PSI. Thus, PSI’s acceptor side is limited in *pgr5*. Because of acceptor side limitation, one could expect more ROS generation at the PSI acceptor side (Tiwari et al., 2016), suggesting that lipid synthesis could serve as a receptor to excess electrons to acclimatize to the abiotic stress. As already reported, oxidative stress causes lipid accumulation under nitrogen stress in microalgae (Yilancioglu et al., 2014).

Transmission electron microscopy (TEM) images of *C. reinhardtii* showed a pronounced increase in vacuoles’ size under high light intensity, particularly in mutant strains (Figure 6). Accumulated autophagy bodies can play an essential role in lipid metabolism. A similar report was observed in nutrient limitations like nitrogen limiting conditions in *C. reinhardtii* (Couso et al., 2016). It has been reported that in stress conditions (like oxidative stress, rapamycin stress, and ER stress), the ATG8 gene was expressed in *C. reinhardtii* (Perez-Martin et al., 2014). In agreement with this hypothesis, we reported that significant expression of ATG8-PE protein was observed under high light conditions by immunofluorescence microscopy. It indicates that ATG8 is a pivotal protein to induce the autophagy process in *C*. *reinhardtii* (Figure 4). An abundance of ATG8-PE protein was increased in mutants due to high light, especially in *pgr5*. It seems an increase in stromal redox poise, which induces autophagy, thereby leading to triacylglycerol (TAG) accumulation. High light stress leads to ROS formation, which activates the autophagy mechanism. Increased ROS was seen in high light conditions; however, this was more prevalent in *pgr*5 mutant when grown in high light, indicating that high light increases oxidative stress and autophagy, which accompanies increased lipid content in the cell.

The autophagy of *C*. *reinhardtii* contains numerous autophagy-related proteins. Among various ATG proteins, ATG8-PE protein is essential for forming autophagosomes (Pérez-Pérez et al., 2016). In this study, we measured the expression level of ATG8-PE protein to evaluate the autophagy role in WT, *pgrl1*, and *pgr5* (Figure 4). ATG8 appeared as a faint band in the WT and mutants (*pgrl1* and *pgr5*) grown under optimal conditions (50 μmol photons m^–2^ s^–1^), and it was increased when cells were exposed to high light. However, lipidated form ATG8-PE was detected in the *pgr5* mutant. Further, TEM confirmed several lytic vacuoles and small vesicles inside the vacuoles in high light treated cells (Figure 6). Under high light vacuoles size was markedly increased abundantly. This supports our hypothesis that light stress causes ROS production, especially in the *pgr5* mutant. In turn, ROS induces autophagy, as reported earlier, leading to an increase in lipid production. Confocal and fluorescent activated cell sorter (FACS) data also suggested an increase in lipid droplet formation under high light in *pgr5* mutant (Figures 5A,D). Continuous light exposure leads to the accumulation of biomass and the cellular over reduction and formation of ROS, which induces autophagy and lipids in microalgae (Shi et al., 2017). Our previous results indicate that PSI and PSII were damaged when cells were grown in high light (Nama et al., 2019; Yadav et al., 2020). Based on this result, we interpret that lipid accumulation in mutant strains could be explained by PSII or PSI being over-reduced, leading to high ROS production. These increased ROS levels act as an autophagy inducer (Heredia-Martinez et al., 2018).

### High Light Induces a High Amount of Lipids

In microalgae, lipid and carbohydrate are the primary energy storage forms and share the common carbon precursors for biosynthesis. Carbon partitioning is essential for the development of biofuels and chemicals. Most carbohydrates are stored as starch and sugars in microalgae. In several algal species, carbohydrate and lipid accumulate, while in some species, carbohydrate level decreases, and TAG increases, suggesting that lipids may be synthesized from carbohydrate degradation. Our results showed that both lipid and carbohydrate content increased in WT and *pgrl1*. However, lipid accumulation precedes carbohydrate accumulation (Figures 5, 7), consistent with the previous studies in the microalga, *Pseudochlorococcum*, under nitrogen stress conditions (Li et al., 2011). However, the carbohydrate content was reduced in *pgr5* mutant under high light growth, explaining that carbohydrate degradation would have been converted to lipids in *pgr5*. Therefore more lipid accumulation was observed. Complex metabolic regulations in the cell may control the accumulation of lipid droplets. The *pgr5* prevents the proton gradient and PSI dependant CET; therefore, the altered NADPH and ATP ratio may switch the metabolic pathways during the photosynthesis process. Some of the metabolic alterations would have converted to lipids, which is necessary to acclimatize to the high light in *pgr5*. Carbohydrate is the dominant sink for carbon storage in *C. reinhardtii* mutant, which lacks cell walls, so lipid synthesis occurs only when the carbon supply exceeds carbohydrate synthesis (Fan et al., 2012). Under saturating light, the *C. reinhardtii* culture induces lipid droplets formation without disrupting growth, while N starvation leads to significantly lower level (Goold et al., 2016).

In our case, a significant accumulation of TAG was observed in high light conditions and the accumulation of TAG is more in *pgr5* (Figure 5). In, *Arabidopsis thaliana* and *C*. *reinhardtii* TAG synthesis originated from multiple types of acyltransferases. The final step of TAG biosynthesis was catalyzed by diacylglycerol acyltransferases (DGAT) and phospholipid diacylglycerol acyltransferase (PDAT) both involved in seed oil accumulation (Zhang et al., 2009). In the present study, we find the regulation of PDAT and DGAT at the protein level. Both the enzymes are accumulated under high light intensity, supporting these enzymes in the synthesis of TAG (Figure 8). Thus, the accumulation of TAG would be either through DGAT or PDAT routes.

### High Light Alters the Fatty Acid Composition in *C. reinhardtii*

The fatty acid composition also plays a significant role in biofuel production. The results of total fatty acids show that the composition and content of fatty acids were different in WT, *pgrl1*, and *pgr5* under normal and high light. The *pgrl1* and *pgr5* have a higher percentage of SFA and MUFA under high light, as shown in Table 1. Palmitic (C16:0), stearic acid (C18:0), oleic acid content (C18:1), and palmitoleic (C16:1) increased in WT, *pgrl1*, and *pgr5* under high light. This result implies high light stress conditions increase SFA and MUFA content in WT, *pgrl1*, and *pgr5* mutant because the excess fatty acids could provide energy sink to the cells. Overall total fatty acid content was enhanced in *pgr5* mutant compared to WT and *pgrl1*. We also observed an increase in the individual TAG band, especially the oleic acid (18:1 ω9c) (Figure 9A). Usually, oleic acid is a source of carbon that requires a functional β-oxidation cycle in which oleic acid is reduced to acetyl-CoA then to sugars or synthesis of other fatty acids or lipids (Graham, 2008).

Interestingly, the thin layer chromatography results show that the membrane lipids have been decreased, which indicates that the degradation of these lipids could have converted to TAG, especially in *pgrl1* and *pgr5* (Supplementary Figure 4). Also, the decreased fatty acids from MGDG and DGDG could have converted to TAG (Figure 9). Similar reports were observed suggesting the recycling of membrane lipids, monogalactosyldiacylglycerol (MGDG), and digalactosyldiacylglycerol (DGDG) into TAG accumulation in *C. reinhardtii* (Abida et al., 2015; Devadasu and Subramanyam, 2021). Therefore, we can interpret that fatty acids are dissociated from glycolipids, which are involved in the Kennedy pathway to synthesize the TAGs through the DGAT enzyme (Yoon et al., 2012).

Our results provided complete evidence that the two mutants, *pgrl1* and *pgr5*, of *C. reinhardtii*, accumulate fatty acid and induced autophagy under high light conditions. The correlation between stress and TAG accumulation in photosynthesis of algae has been known for a century. Under high light conditions, these mutant’s fatty acid compositions may be an important study for biotechnological applications. It suggests that one possible reason for the increase in lipid content may be the unbalanced redox state of the cells, leading to the generation of ROS. Therefore, the increased ROS levels in *pgr5* cells could play a dual role as signals to activate lipid biosynthesis and autophagy inducers to start recycling cellular components to fuel lipid production in this mutant (Tran et al., 2019). Therefore, *pgrl1* and *pgr5* of *C. reinhardtii* would be appropriate for producing high yield lipids (Lipids/TAG). Hence, our study could offer an essential value: microalgae-based lipid production can be promoted by applying various feedstocks to biodiesel and animal feed.

## Data Availability Statement

The original contributions presented in the study are included in the article/Supplementary Material, further inquiries can be directed to the corresponding author.

## Author Contributions

NC, ED, and RMY carried out the experiments, analyzed the data, and drafted the manuscript. RS designed the experiments, drafted the manuscript, and secured the fund. All authors approved the final version before submission.

## Conflict of Interest

The authors declare that the research was conducted in the absence of any commercial or financial relationships that could be construed as a potential conflict of interest.

## Publisher’s Note

All claims expressed in this article are solely those of the authors and do not necessarily represent those of their affiliated organizations, or those of the publisher, the editors and the reviewers. Any product that may be evaluated in this article, or claim that may be made by its manufacturer, is not guaranteed or endorsed by the publisher.

## Supplementary Material

Supplementary Figure 1 Quantification of localization of ATG8 in *C. reinhardtii* cells under high light conditions from microscope image. Quantification of number of ATG8 from 50 μmol photons m^–2^ s^–1^ and 500 μmol photons m^–2^ s^–1^. Quantification was carried out from single cell with three individual cultures. Data are expressed as mean ± SD of 3 replicates. Statistical significance was analyzed using one-way ANOVA with Tukey test and *p*-value obtained are indicated (^***^*p* < 0.001 and ^**^*p* < 0.01).

Supplementary Figure 2 Lipid droplets were identified through confocal microscopy. Cells were grown under 50 μmol photons m^–2^ s^–1^ and 500 μmol photons m^–2^ s^–1^ were stained with Nile Red (5 μM/mL) for lipid droplets in *C. reinhardtii* strains, WT, *pgrl1*, and *pgr5*. The images were collected with three individual measurements for all the conditions. Scale bar = 20 μm.

Supplementary Figure 3 Neutral lipid content is quantified from the cells grown under normal and high light. Neutral lipid content was qualitatively measured with Nile Red fluorescence from the cells WT, *pgrl1*, and *pgr5* grown with normal (50 μmol photons m^–2^ S^–1^) and high (500 μmol photons m^–2^ S^–1^) light condition stained with Nile Red on days 1–4. The measurements have been done with a microplate reader. Three biological experiments were done (*n* = 3). In ‘*x*’ axis WT, *pgrl1* and *pgr5* 50; WT, *pgrl1* and *pgr5* 500, represents 50 and 500 μmol photons m^–2^ s^–1^. Statistical significance was analyzed using one-way ANOVA, and subsequent Tukey’s *post hoc t-*test and *p*-value obtained are indicated (^***^*p* < 0.001).

Supplementary Figure 4 Ultrastructure analysis of *C. reinhardtii* cells during normal (50 μmol photons m^–2^ s^–1^) and high light (500 μmol photons m^–2^ s^–1^). Transmission electron micrographs of *C. reinhardtii* cells WT and mutants *pgrl1* and *pgr5* grown under photoheterotrophic conditions under 50 μmol photons m^–2^ s^–1^ and 500 μmol photons m^–2^ s^–1^. Representative images were taken after the 3rd day of growth. Three independent experiments were conducted from each sample (*n* = 3).

Supplementary Figure 5 Alteration of membrane lipid and TAG in *C. reinhardtii* cells grown under normal and high light conditions. **(A)** TLC analysis showing TAG accumulation of *C. reinhardtii* cells WT and mutants *pgrl1* and *pgr5* strains under 50 μmol photons m^–2^ s^–1^ and 500 μmol photons m^–2^ s^–1^. Three independent experiments were conducted for all samples (*n* = 3). **(B)** Separation of polar lipids by TLC from *C. reinhardtii* strains WT, *pgrl1*, and *pgr5* under 50 μmol photons m^–2^ s^–1^ and 500 μmol photons m^–2^ s^–1^. Polar lipids and TAG was visualized by iodine staining. Three independent experiments were conducted for all samples (*n* = 3). WT, *pgrl1* and *pgr5* 50, WT, *pgrl1* and *pgr5* 500, represents 50 and 500 μmol photons m^–2^ s^–1^. TAG, triacylglycerol. DGDG, digalactosyldiacylglycerol; MGDG, monogalactosyldiacylglycerol; PC, phosphatidylcholine; PE, phosphatidylethanolamine; PG, phosphatidylglycerol; PI, phosphatidylinositol; SQDG, sulfoquinovosyldiacylglycerol.
