# Supplementary figures and images for: Autophagy Induced Accumulation of Lipids in pgrl1 and pgr5 of Chlamydomonas reinhardtii Under High Light
Source: Front Plant Sci. 2022 Jan 25;12:752634. doi: 10.3389/fpls.2021.752634 (PMC8821104; doi:10.3389/fpls.2021.752634)

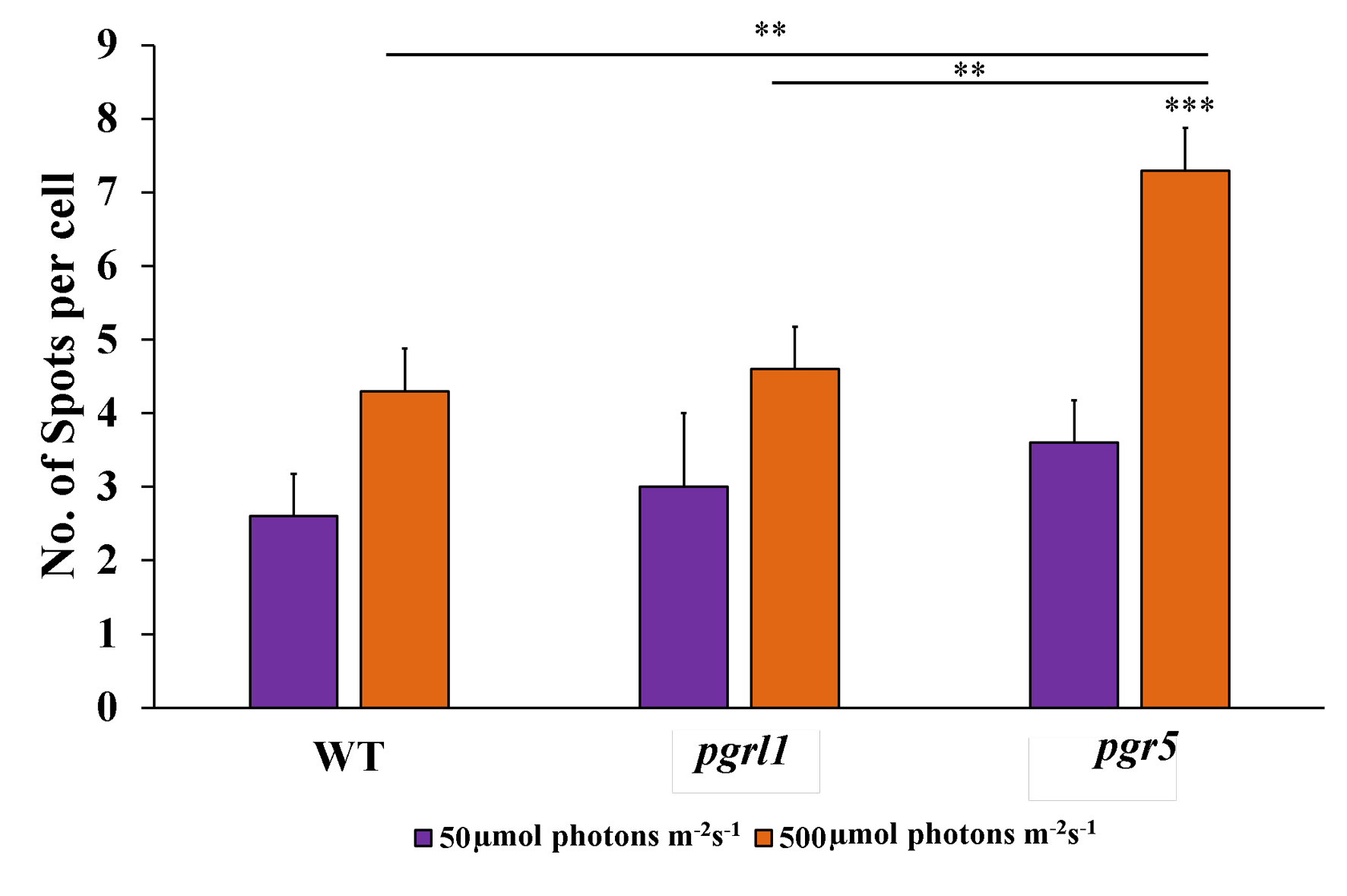

Supplement: Supplementary Figure 1 — Quantification of localization of ATG8 in C. reinhardtii cells under high light conditions from microscope image. Quantification of number of ATG8 from 50 μmol photons m–2 s–1 and 500 μmol photons m–2 s–1. Quantification was carried out from single cell with three individual cultures. Data are expressed as mean ± SD of 3 replicates. Statistical significance was analyzed using one-way ANOVA with Tukey test and p-value obtained are indicated (***p < 0.001 and **p < 0.01). [file Image_1.TIF]

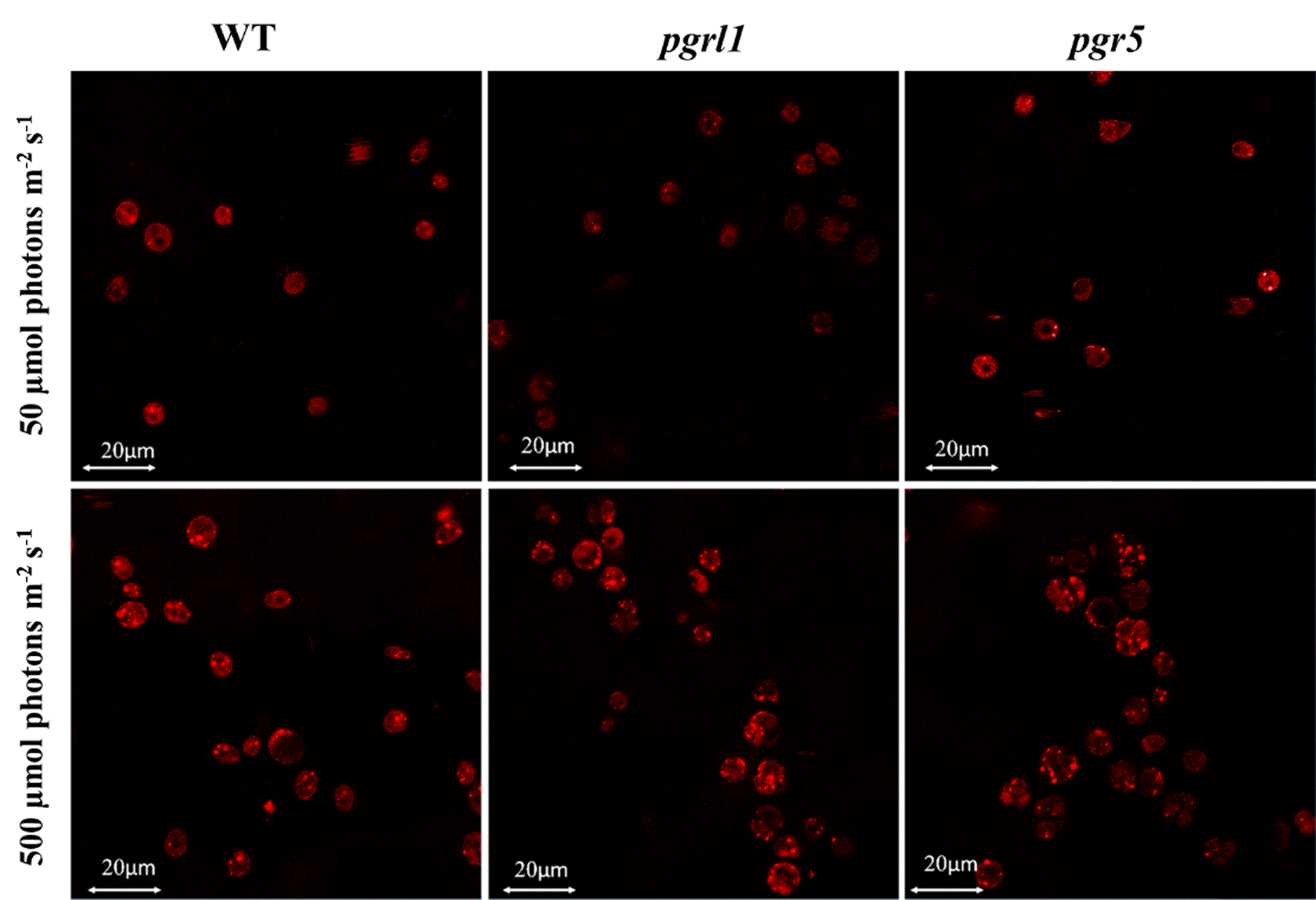

Supplement: Supplementary Figure 2 — Lipid droplets were identified through confocal microscopy. Cells were grown under 50 μmol photons m–2 s–1 and 500 μmol photons m–2 s–1 were stained with Nile Red (5 μM/mL) for lipid droplets in C. reinhardtii strains, WT, pgrl1, and pgr5. The images were collected with three individual measurements for all the conditions. Scale bar = 20 μm. [file Image_2.TIF]

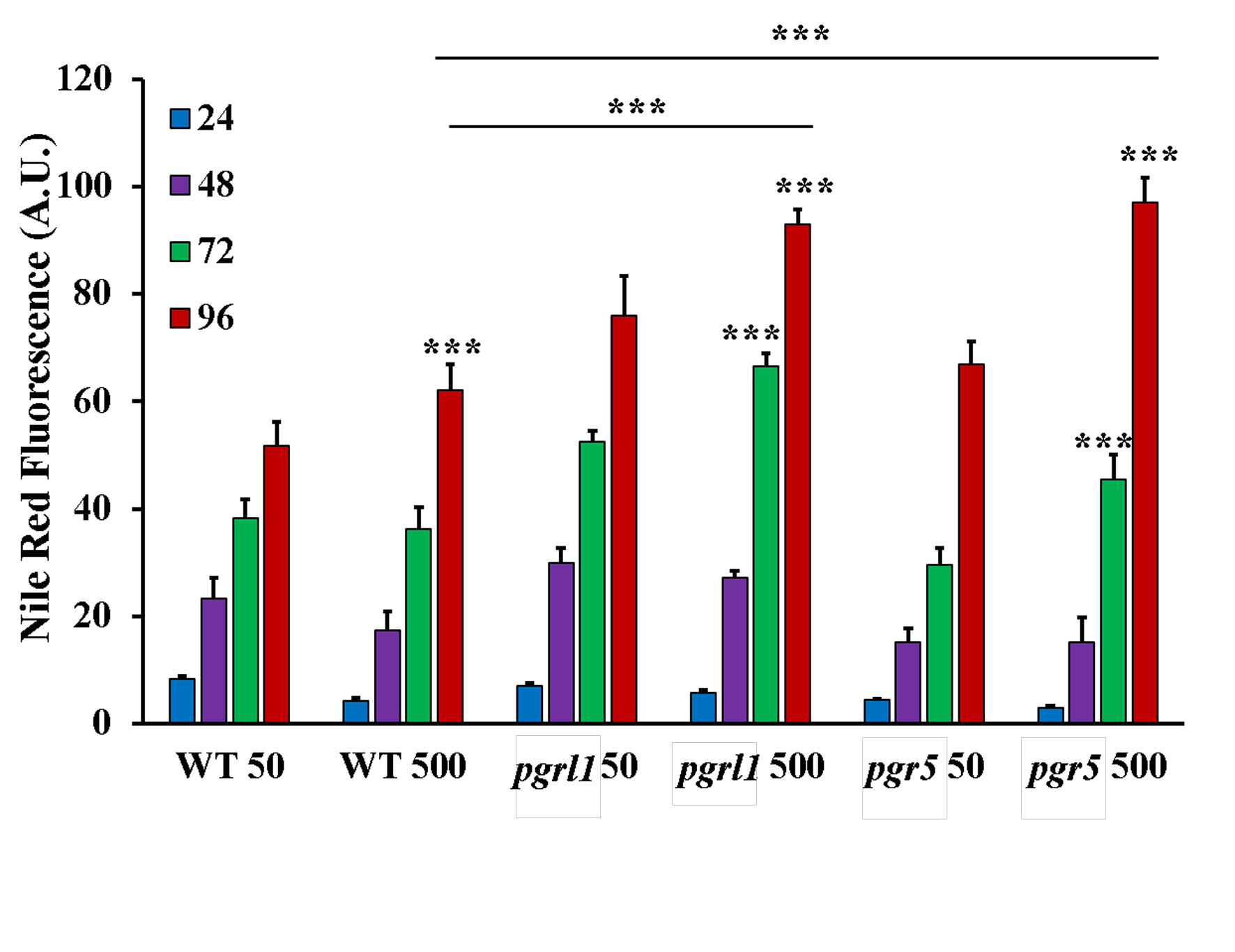

Supplement: Supplementary Figure 3 — Neutral lipid content is quantified from the cells grown under normal and high light. Neutral lipid content was qualitatively measured with Nile Red fluorescence from the cells WT, pgrl1, and pgr5 grown with normal (50 μmol photons m–2 S–1) and high (500 μmol photons m–2 S–1) light condition stained with Nile Red on days 1–4. The measurements have been done with a microplate reader. Three biological experiments were done (n = 3). In ‘x’ axis WT, pgrl1 and pgr5 50; WT, pgrl1 and pgr5 500, represents 50 and 500 μmol photons m–2 s–1. Statistical significance was analyzed using one-way ANOVA, and subsequent Tukey’s post hoc t-test and p-value obtained are indicated (***p < 0.001). [file Image_3.TIF]

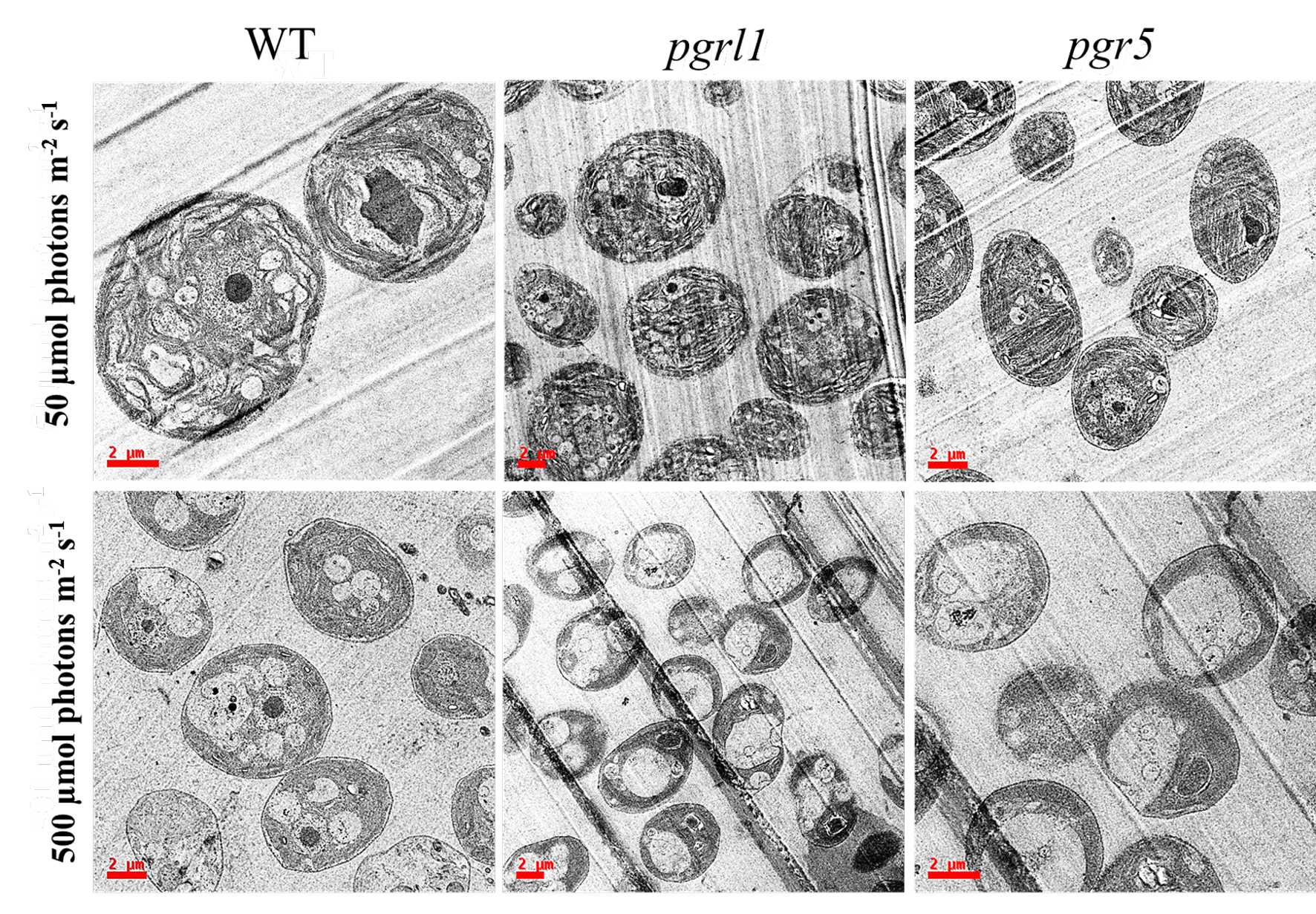

Supplement: Supplementary Figure 4 — Ultrastructure analysis of C. reinhardtii cells during normal (50 μmol photons m–2 s–1) and high light (500 μmol photons m–2 s–1). Transmission electron micrographs of C. reinhardtii cells WT and mutants pgrl1 and pgr5 grown under photoheterotrophic conditions under 50 μmol photons m–2 s–1 and 500 μmol photons m–2 s–1. Representative images were taken after the 3rd day of growth. Three independent experiments were conducted from each sample (n = 3). [file Image_4.TIF]

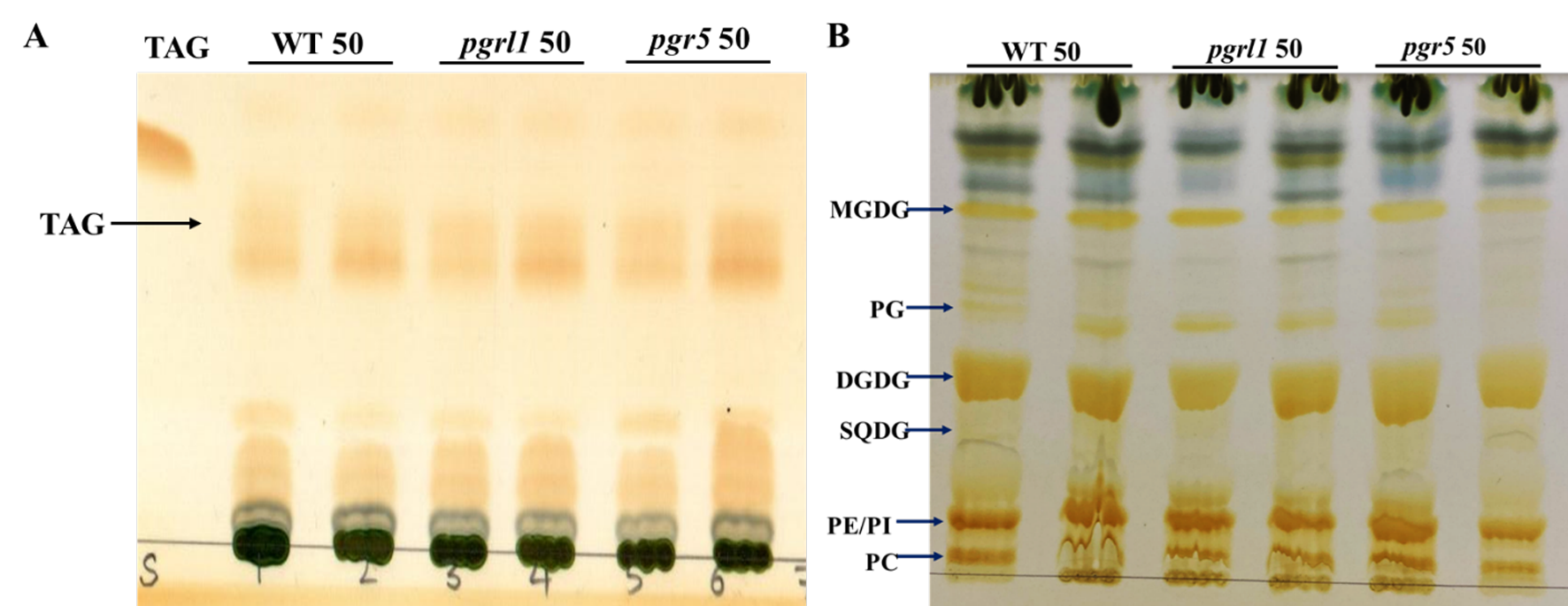

Supplement: Supplementary Figure 5 — Alteration of membrane lipid and TAG in C. reinhardtii cells grown under normal and high light conditions. (A) TLC analysis showing TAG accumulation of C. reinhardtii cells WT and mutants pgrl1 and pgr5 strains under 50 μmol photons m–2 s–1 and 500 μmol photons m–2 s–1. Three independent experiments were conducted for all samples (n = 3). (B) Separation of polar lipids by TLC from C. reinhardtii strains WT, pgrl1, and pgr5 under 50 μmol photons m–2 s–1 and 500 μmol photons m–2 s–1. Polar lipids and TAG was visualized by iodine staining. Three independent experiments were conducted for all samples (n = 3). WT, pgrl1 and pgr5 50, WT, pgrl1 and pgr5 500, represents 50 and 500 μmol photons m–2 s–1. TAG, triacylglycerol. DGDG, digalactosyldiacylglycerol; MGDG, monogalactosyldiacylglycerol; PC, phosphatidylcholine; PE, phosphatidylethanolamine; PG, phosphatidylglycerol; PI, phosphatidylinositol; SQDG, sulfoquinovosyldiacylglycerol. [file Image_5.TIF]
