# Supplementary material for: Autophagy Induced Accumulation of Lipids in pgrl1 and pgr5 of Chlamydomonas reinhardtii Under High Light
Source: Front Plant Sci. 2022 Jan 25;12:752634. doi: 10.3389/fpls.2021.752634 (PMC8821104; doi:10.3389/fpls.2021.752634)
Supplement: Supplementary file 6 [file Data_Sheet_1.docx]

**STable 1** Optical density of *C.reinhardtii* under normal (50 µmol photons m^-2^s^-1^) and high light (500 µmol photons m^-2^s^-1^) conditions. n=3. WT, *pgrl* and *pgr5* 50; WT, *pgrl* and *pgr5* 500, represents 50 and 500 µmol photons m^-2^ s^-1^.

| Optical density | | | | | | |
| --- | --- | --- | --- | --- | --- | --- |
| Hrs | WT 50 | WT500 | *pgrl1* 50 | *pgrl1* 500 | *pgr5* 50 | *pgr5* 500 |
| 0 | 0.019±0.002 | 0.024±0.0014 | 0.023±.001 | 0.0226±.001 | 0.026±.009 | 0.023±.0008 |
| 24 | 0.28±0.41 | 0.216±.0051 | 0.051±.003 | 0.0223±.003 | 0.14467±.0035 | 0.163±.0051 |
| 48 | 0.36±0.05 | 0.68±.0055 | 0.167±.001 | 0.115±.046 | 0.306±.0096 | 0.172±.0051 |
| 72 | 0.79±0.005 | 1.08±.0545 | 0.661±.002 | 0.553±.006 | 0.846±.0464 | 0.719±.017 |
| 96 | 1.06±0.08 | 1.34±.0475 | 1.238±.092 | 1.179±.121 | 1.162±.0248 | 1.301±.055 |
